# Genetic Evidence for Bacterial Chemolithoautotrophy Based on the Reductive Tricarboxylic Acid Cycle in Groundwater Systems

**DOI:** 10.1264/jsme2.ME11274

**Published:** 2012-02-22

**Authors:** Albin Alfreider, Carsten Vogt

**Affiliations:** 1 Institute of Ecology, University of Innsbruck, Technikerstr. 25, 6020 Innsbruck, Austria; 2 Department of Isotope Biogeochemistry, Helmholtz Centre for Environmental Research–UFZ, Permoserstraße 15, 04318 Leipzig, Germany

**Keywords:** Groundwater, chemoautotroph, CO_2_ fixation, reverse tricarboxylic acid cycle

## Abstract

Geologically and chemically distinct aquifers were screened for the presence of two genes coding for key enzymes of the reverse tricarboxylic acid (rTCA) cycle in autotrophic bacteria, 2-oxoglutarate : ferredoxin oxidoreductase (*oorA*) and the beta subunit of ATP citrate lyase enzymes (*aclB*). From 42 samples investigated, *aclB* genes were detected in two and *oorA* genes in six samples retrieved from polluted and sulfidic aquifers. *aclB* genes were represented by a single phylotype of almost identical sequences closely affiliated with chemolithoautotrophic *Sulfurimonas* species. In contrast, sequences analysis of *oorA* genes revealed diverse phylotypes mainly related to sequences from cultivation-independent studies.

To date, six mechanisms are known by which autotrophic organisms fix carbon (7). A frequent CO_2_ fixation strategy in chemolithoautotrophic bacteria occurs via the Calvin-Benson-Bassham cycle (6). The enzyme responsible for the actual fixation of CO_2_ is ribulose-1,5-bisphosphate carboxylase/oxygenase (RubisCO) and genes coding for the large subunit of RubisCO are often used to analyse chemolithoautotrophs in cultivation-independent environmental studies. Several investigations have been performed in groundwater systems, which revealed the widespread occurrence of diverse forms of RubisCO and hence autotrophic bacteria assimilating CO_2_ via the Calvin cycle pathway in the subsurface (2, 4).

The reductive tricarboxylic acid cycle (rTCA) is, in addition to the Calvin cycle, a carbon fixation pathway which is frequently found in prokaryotes: The rTCA cycle appears to operate in phylogenetically diverse autotrophic bacteria (*Aquificae*, *Nitrospirae*, *Chlorobi*, *Proteobacteria*), including genera of anoxic phototrophic bacteria, sulfate-reducing bacteria and hyperthermophilic hydrogen-oxidizing bacteria (7, 17). Although special biochemical adaptations to oxic conditions are known, this pathway involves enzymes that are sensitive to oxygen. As a result, rTCA-based CO_2_ fixation is mostly found in anaerobes or microaerophiles (7). Cultivation-independent investigation based on the detection of genes coding for key enzymes in the rTCA pathway were mainly performed in hydrothermal vent systems and thermal springs (*e.g.* 10, 14, 18, 27). The results of these studies suggest that the rTCA cycle is a major autotrophic pathway in thermal and sulfidic environments and molecular evidence also suggests that the Calvin cycle is often less prevalent in these ecosystems. On the other hand, autotrophy based on the rTCA cycle is not restricted to hot ecosystems. Several isolates from bacterial enrichment studies and environmental investigations performed with samples from terrestrial ecosystems, activated sludge, saline lakes, sulfidic caves, nonvent marine systems and an estuary revealed the presence of taxonomically diverse bacteria potentially using the rTCA cycle for CO_2_ fixation (*e.g.* 8, 11, 21, 23, 24, 29, 31, 37 and references therein); however, their ecological significance in these habitats is often not well understood.

To our knowledge, the distribution and diversity of autotrophic bacteria assimilating CO_2_ via the rTCA pathway has not been analyzed in groundwater systems. In the present study, 42 groundwater samples from a variety of aquifers were screened for the presence of two genes coding for key enzymes of the rTCA cycle: the alpha subunit of 2-oxoglutarate : ferredoxin oxidoreductase enzymes (*oorA* genes) and the beta subunit of ATP citrate lyase enzymes (*aclB* genes). Amplification products, which were successfully retrieved from six samples, were used to construct clone libraries and selected positive clones were sequenced in order to evaluate their phylogenetic relationships.

Successful amplification of *aclB* and *oorA* genes was achieved with samples of two groundwater systems, both contaminated with organics (Table 1): (a) At an on-site underground facility close to the city of Bitterfeld, 50 km north of Leipzig (Germany). Samples were taken from a horizontal well (19.5 meter below surface) located in a quaternary aquifer and from a column filled with original aquifer material. The groundwater is rich in sulfate and is slightly sulfidic; oxygen, nitrate and nitrite are below the detection limit (Table 1). Chlorobenzene is the main contaminant (35); (b) In groundwater influenced by activities of a former hydrogenation plant close to the city of Zeitz (Saxonia, Germany), 40 km southwest of Leipzig, where benzene production caused massive groundwater contamination. Naturally existing sulfate was found to be the main electron acceptor for microbial oxidation reactions in the central parts of the plume. Nitrate was detected on the fringes of the plume, indicating nitrate reduction as an additional important electron-accepting process at the site. Although oxygen concentrations were not measured *in situ*, the presence of sulfide in the investigated sampling sites indicates anoxic, sulfidic conditions in the aquifer (Table 1).

Groundwater systems, where rTCA cycle genes were not detected, included sampling campaigns performed in non-polluted and shallow groundwater systems (Salzburg, Austria), a methyl tert-butyl ether (MTBE), BTEX and ammonia-polluted groundwater remediation test site near Leuna (Germany) and from deep subsurface samples retrieved by exploratory drilling along the projected Brenner Base Tunnel (Tyrol, Austria) (Table 1); these sampling sites are described in more detail elsewhere (4).

For DNA extraction, volumes between 300 to 500 mL groundwater were concentrated on filters (pore size 0.22 μm; Durapore, Millipore, Bedford, MA, USA) and immediately frozen until analysis. Aquifer sediments from the underground columns in Zeitz and Bitterfeld were sampled with sample lances, immediately transferred to an anoxic jar (Anaerocult A; Merck, Germany), transported at 4°C and stored at −20°C before further processing. DNA extraction of groundwater and sediment samples was performed with the FastDNA Spin Kit for soil (Qbiogene, Carlsbad, CA, USA) according to the manufacturer’s instructions and as described by Alfreider *et al.*(4). Two sets of oligonucleotide primers were used for PCR amplification of *aclB* and *oorA* gene fragments as described in Campbell *et al.*(9) and Campbell and Cary (10).

PCR products of appropriate length were cloned into a pDrive Cloning Vector (Qiagen, Valencia, CA, USA) according to the protocols provided by the manufacturer and as described in Alfreider *et al.*(4). Plasmid DNA was isolated (Qiagen plasmid kit; Qiagen), and clones were screened for the presence of inserts by PCR using vector-specific primers. Full PCR fragments were sequenced with capillary genetic analyzers (ABI 3730 or ABI3700; Applied Biosystems, Foster City, CA, USA) by an external sequencing facility (Macrogen, Seoul, Korea). Closest relatives to *oorA* and *aclB* nucleotide sequences and deduced amino acid sequences were obtained using NCBI’s sequence similarity search tools BLASTN and BLASTP (5), the IMG/M data management and analysis system for metagenomes (26) and CAMERA metagenomic resources (30). Deduced amino acids were aligned using Clustal W, as provided by MEGA 4 software followed by visual inspection of the alignment (33). Neighbor-joining trees applying gamma distribution as the distance method were computed with the MEGA 4 software package. Bootstrap analysis (1,000 replicates) was used to obtain confidence estimates for tree topology. Sequence data have been submitted to GenBank databases under accession numbers JF794648–JF794655 for *aclB* genes and JF794656–JF794687 for *oorA* genes.

PCR screening of 42 samples revealed the presence of genes coding for oxoglutarate : ferredoxin oxidoreductase enzymes in six samples and genes coding for ATP citrate lyase enzymes were detected in two samples (Table 1). PCR products were cloned and positive clones with the proper insert length were selected for sequence analysis. ATP citrate lyase genes were detected in sampling station Bitterfeld B5 with seven identical sequences (based on deduced amino acid sequences) and one sequence was retrieved from the groundwater well Zeitz Z5 (Fig. 1). Phylogenetic analysis revealed that these sequences formed a unique cluster and showed 90 to 91% amino acid sequence similarities with ɛ-proteobacterial *aclB* sequences. These closest relatives include deep-sea hydrothermal *Sulfurimonas* isolates (32), deep-sea hydrothermal chemolithoautotrophic isolates of *ɛ-Proteobacteria*, epibiontic bacteria associated with the hydrothermal vent polychaete *Alvinella pompejana*(9) and free-living microorganisms obtained from deep-sea hydrothermal vents (10). However, the sequence with the highest similarity to *aclB* genes obtained from samples B5 and Z5 was found in the non-vent bacterium *Sulfurimonas denitrificans*, a mesophilic sulfur oxidizer isolated from coastal marine sediments (31, 34).

In contrast to the *aclB* genes, the analysis of sequences coding for 2-oxoglutarate:ferredoxin oxidoreductase enzymes showed a high degree of diversity. The amino acid sequences deduced from *oorA* genes sequences retrieved from horizontal well B5 (sampling station Bitterfeld) revealed three phylotypes (Fig. 2). Sequence Bitterfeld B5-A5 was affiliated with several sequences obtained from sampling stations Z5, Z6 and Z11 of the Zeitz aquifer. Sequence Bitterfeld B5-2 and the closely related sequence Zeitz Z6-B21 are affiliated with *Syntrophobacter fumaroxidans* str. MPOB (89% sequence similarity). Two Bitterfeld B5 clones (B5-A9 and B5-A6) were clearly separated from all other *oorA* genotypes retrieved in this study (Fig. 2). Both sequences clustered with *oorA* genes derived from deep-sea hydrothermal chemolithoautotrophic isolates and cultivation-independent studies. *Sulfurimonas denitrificans* was the closest relative with 94% amino acid similarity. As already mentioned above, *Sulfurimonas denitrificans* and other *Sulfurimonas* species were also the closest relatives of all *aclB* sequences observed at sampling station Bitterfeld B5. *S. denitrificans*, an obligate chemolithoautotrophic *ɛ-Proteobacterium*, is a metabolically versatile bacterium with the potential to use a variety of compounds as electron donors, *e.g.* reduced sulfur compounds, hydrogen or formate (31). *S. denitrificans*, which was originally isolated from coastal marine sediments (34), is also suggested to be a key player in the nitrogen cycle in the Baltic Sea, responsible for chemolithoautotrophic denitrification (8, 13). In addition to nitrate, *S. denitrificans* can use inorganic sulfur compounds or oxygen as electron acceptors (31).

The *oorA* sequences derived from the sampling stations Zeitz were widespread along the phylogenetic tree. Most sequences were not closely affiliated with known *oorA* genotypes deposited in public databases and therefore these genotypes were also distantly related to cultivated representatives. Consequently, it would be too speculative to deduce ecophysiological characteristics from these sequences. Comparison with deduced amino acid sequences retrieved from sequences of public databases showed closest similarities ranging between 76% and 92%. For example one cluster of *oorA* sequences from groundwater well Zeitz Z11 (clones 31, B46, B47, B50) was affiliated (84%–88% deduced amino acid identity) with an *oorA* gene obtained from a metagenomic study of a hypersaline mat from Guerrero Negro, Mexico, which is characterized by steep physicochemical gradients on the millimeter scale (22). Several *oorA* genes retrieved in our study were affiliated with sequences of different metagenomic investigations, including a thermophilic terephthalate-degrading microbial community growing at around 55°C that was developed in a lab-scale bioreactor (IMG/M database, not published), *oorA* genes from a highly stable reductive dechlorinating bioreactor (IMG/M database, not published), microbial communities from Yellowstone hot springs and hot spring pools (IMG/M database, not published) and symbionts from the marine oligochaete *Olavius algarvensis*(38). Summarizing the phylogenetic analysis based on genes coding for 2-oxoglutarate:ferredoxin oxidoreductase enzymes revealed that the majority of sequences were affiliated with *oorA* sequences derived from cultivation independent studies, generally thermal ecosystems and/or environments that are characterized by distinct concentration gradients and different redox states of bio-geochemical important elements supporting reverse tricarboxylic acid cycle as the autotrophic pathway.

The presence of phylogenetically distant *oorA* sequences, however, suggests the occurrence of *oorA* phylotypes coding for 2-oxoglutarate : ferredoxin oxidoreductase enzymes involved in other (non rTCA-related) biochemical processes. A reaction that could specifically be of interest at study site Zeitz is the potential role of 2-oxoglutarate : ferredoxin oxidoreductase for aromatic ring reduction (12), since field site Zeitz is contaminated with BTEX compounds with benzene as the main pollutant. Degradation of benzene and toluene under sulfate-reducing conditions has been demonstrated at the site (36), and some of the *oorA* sequences are affiliated with sequences detected in sulfate-reducing or putative syntrophic bacteria (Fig. 2) that are potentially involved in anaerobic BTEX degradation.

To our knowledge, this is the first comprehensive study demonstrating the existence of bacteria with genetic potential using a pathway other than the Calvin-Benson-Bassham cycle for CO_2_ fixation in groundwater ecosystems. From earlier investigations based on RubisCO analysis we have learned that in samples retrieved from the same groundwater sampling sites, the Calvin cycle is widely distributed (in 85% of 48 samples) and used by diverse obligate and facultative chemolithoautotrophic *Proteobacteria* for CO_2_ fixation (4). In the present study, genes for the rTCA cycle were only found in six samples at two sampling locations, with groundwater characterized by anoxic and sulfidic conditions and contaminated with organics. One obvious reason for the limited distribution is the properties of certain enzymes in the rTCA cycle, which are sensitive to oxygen. As a result, the rTCA cycle is only found in anaerobes or microaerophiles. So far, the reductive TCA cycle has been believed to be the main strategy for chemolithoautotrophic CO_2_ fixation in hydrothermal vents and other hot ecosystems. The microbial communities in these habitats are usually dominated by autotrophic *ɛ-Proteobacteria* and *Aquificales*, which generally fix CO_2_ via the rTCA pathway (10, 17, 27). CO_2_ fixation based on the rTCA pathway was also shown for a small number of bacterial representatives phylogentically related to *Chlorobiales*, *δ-Proteobacteria* and magnetotactic cocci affiliated with the class of *α-Proteobacteria*(37), but their environmental importance has yet to be determined.

Previous studies at sampling sites Zeitz and Bitterfeld, focusing on the bacterial composition based on 16S rDNA analysis, revealed the dominance of *β-Proteobacteria* and a broad diversity of other phyla (1, 3). Samples from the Bitterfeld site revealed two clone sequences which were phylogentically classified into the class of *ɛ-Proteobacteria*. Interestingly, the 16S rDNA sequence of one of these clones (RA9C8) was affiliated with the facultatively anaerobic, chemolithoautotrophic, sulfur-oxidizing bacterium *Sulfuricurvum kujiense*(20) and representatives of the genus *Sulfurimonas*, including *S. denitrificans* and *S. autotrophica* (data not shown). A recent study also revealed 16S rDNA sequences related to *ɛ-Proteobacteria* at field site Zeitz (19). A phylotype affiliated to the genus *Sulfurovum* was found to be abundant in a column system operating under close to *in situ* conditions (19); this phylotype has been shown to be involved in anaerobic benzene degradation (15). Furthermore, a phylotype closely related to *S. denitrificans* has been recently identified in nitrate-dependent sulfide-oxidizing enrichment cultures prepared from groundwater from field site Zeitz.

The lifestyle of the versatile *ɛ-Proteobacteria* in organics-contaminated sulfidic sites is not well understood and should be the focus of future studies. Some members of this taxonomic group are autotrophic, using *e.g.* reduced sulfur compounds or hydrogen as electron donors, oxygen, nitrate or metals as electron acceptors, and fixing carbon via the rTCA cycle. In contrast, *Sulfurspirillum* or *Arcobacter* species have been described for fermentative metabolism (25). Recently, phylotypes affiliated mainly to the genus *Sulfuricurvum* were found to be abundant in toluene-degrading sulfate-reducing enrichment cultures prepared from tar-oil contaminated aquifer sediment samples; their function remained unclear (28). Notably, Hubert *et al.*(16) observed that similar phylotypes were dominant in oil formation waters from a Canadian oil sand reservoir; the authors speculate that some of these might use also sulfur-containing crude oil compounds as carbon and/or sulfur sources.

## Figures and Tables

**Fig. 1:**
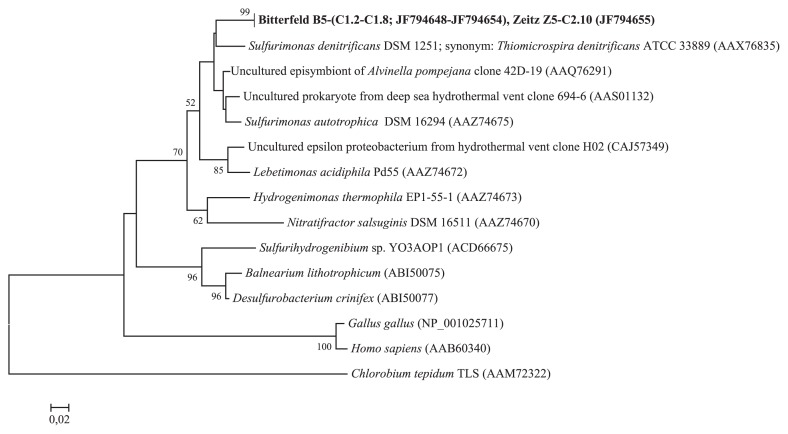
Neighbor-joining phylogenetic tree calculated from deduced amino acids of *aclB* sequences obtained in this study (shown in bold) and public databases. GenBank accession numbers of sequences are given in parentheses. Bootstrap values are shown as percentages of 1,000 replicates and values over 50% are indicated on nodes.

**Fig. 2:**
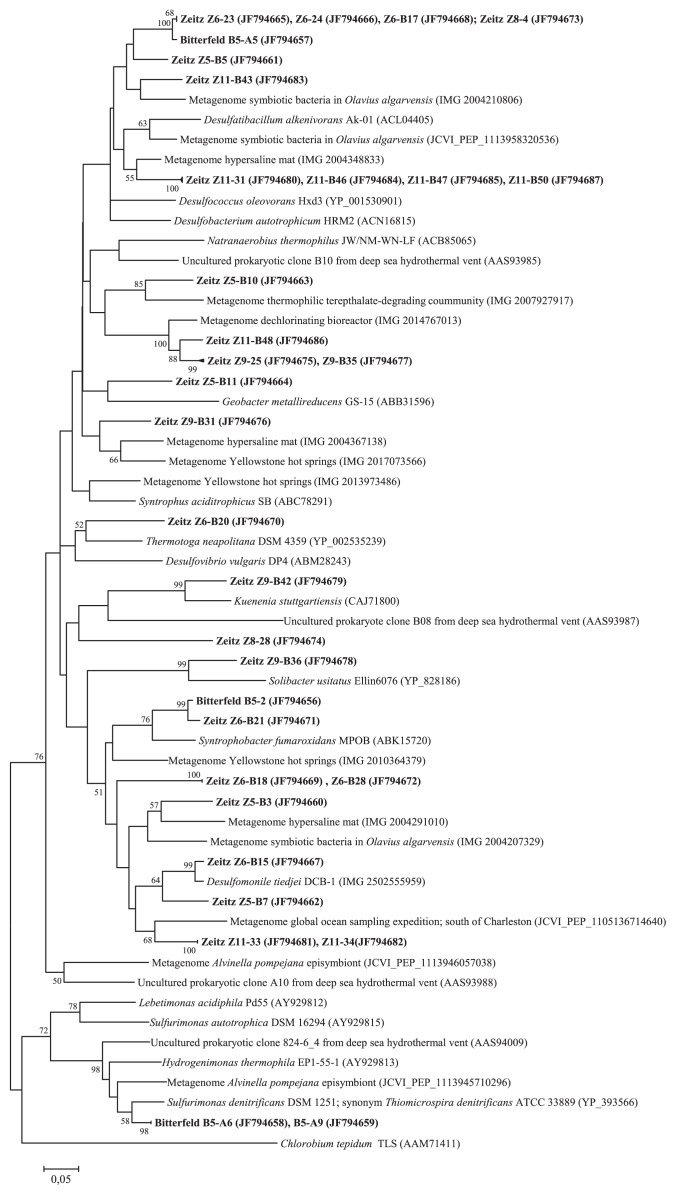
Neighbor-joining phylogenetic tree calculated from deduced amino acids of *oorA* sequences obtained in this study (shown in bold) and public databases. Accession numbers of sequences are given in parentheses. Reference sequences from metagenomic libraries were obtained from the Joint Genome Institute database (IMG) and CAMERA database (JCVI_PEP_). Bootstrap values are shown as percentages of 1,000 replicates and values over 50% are indicated on nodes.

**Table 1:** Description of samples and detection of *aclB* and *oorA* genes based on PCR amplification

Sampling site	Depths (mbs)	Oxygen mg L^−1^	Sulfide mg L^−1^	Sulfate mg L^−1^	Ammonium mg L^−1^	Nitrate mg L^−1^	Nitrite mg L^−1^	Main pollutant	*aclB*	*oorA*
Municipal groundwater Salzburg (5 samples)										
Groundwater samples (S1, S2, S3, S5, S6)	2.9–10.1	3.6–8.5	b.d.	7.8–31.3	b.d.	2.5–39	b.d.	Non-polluted	−	−
Reactor facility Bitterfeld (5 samples)										
Aquifer sediment samples (B1, B2, B3, B4)	column	b.d.	n.d.	681–871	5–5.4	b.d.	b.d.	Chlorobenzene	−	−
Groundwater inflow from horizontal well 1 (B5)	23	b.d.	b.d.–1	714	5.3	b.d.	b.d.	Chlorobenzene	+	+
Pilot Plant Leuna (6 samples)										
Groundwater samples (L1–L6)	channels	0.4–1.6	b.d.–0.04	473–601	45.6–61.4	b.d.–34.9	b.d.–1.7	MTBE	−	−
Test field Zeitz (10 samples)										
Pumice sample (Z1)	column	n.d.	23.5	332	2.5	n.d.	n.d.	Benzene	−	−
Coarse sand sample (Z2)	column	n.d.	12.2	391	1.9	n.d.	n.d.	Benzene	−	−
Groundwater sample—column inflow (Z3)		n.d.	11.1	391	2.2	n.d.	n.d.	Benzene	−	−
Groundwater sample (Z4)	13.9	n.d.	0.6	n.d	1.1	n.d.	n.d.	Benzene	−	−
Groundwater sample (Z5)	19.1	n.d.	8.1	n.d.	5.5	n.d.	n.d.	Benzene	+	+
Groundwater sample (Z6)	42	n.d.	4.6	n.d.	3.6	n.d.	n.d.	Benzene	−	+
Groundwater sample (Z7)	47	n.d.	4	n.d.	3.7	n.d.	n.d.	Benzene	−	−
Groundwater sample (Z8)	53	n.d.	0.7	n.d.	3.3	n.d.	n.d.	Benzene	−	+
Groundwater sample (Z9)	20	n.d.	0.7	n.d.	7.7	n.d.	n.d.	Benzene	−	+
Groundwater sample (Z11)	11.5	n.d.	0.3	n.d.	b.d.	n.d.	n.d.	Benzene	−	+
Deep subsurface—Brenner base tunnel (16 samples)										
Groundwater samples (V1–V5; P1, P2; N; A1–A3; E1–E3; M1, M2)	48–780	n.d.	b.d.	14–1296	b.d.–0.24	b.d.–2.56	b.d.–0.04	Non-polluted	−	−

b.d.: below detection limit

n.d.: not determined

+ PCR amplification product (samples underlined)

− no PCR amplification product
